# The Relationship Between Mental Health and Social Solidarity Among Apartment Residents in Shahrekord, Iran

**Published:** 2012

**Authors:** Forouzan Ganji, Afsaneh Nekooee, Faranak Safdari, Neda Parvin, Akbar Shafei, Hanife Ganji

**Affiliations:** 1 Community Medicine Department, Shahrekord University of Medical Sciences, Shahrekord, Iran

**Keywords:** Apartment resident, Individuals’ relations, Mental health, Social solidarity

## Abstract

**Objective**: To examine the relationship between psychological well-being and social solidarity of apartment residents in Shahrekord, Iran.

**Methods:** A sample of 200 apartment dwellers was selected randomly. Fessler Social Solidarity Inventory and General Health Questionnaire were used to gather data.

**Results:** Using partial correlation test and having controlled the effect of age, sex and education, we found significant relationship between mental health and social solidarity (r = 0.47; p= 0.023). After controlling education and marital status, it was also revealed that women were in a better solidarity situation compared to men (p< 0.05).

**Conclusion: **There is a relation between the mental health and social solidarity of apartment residents in Sharekord. Good mental health accompanied with better social solidarity.

## Introduction

In recent decades, an insatiable demand has been made for urban housing due to the growth of population in Iran, especially in urban areas. According to estimates, about 22 million houses are needed for 100 to 120 million Iranians by the year 2021 (1). 

This high demand for housing has led to multifarious problems for city dwellers. The vertical growth of cities due to multi-storey residential apartment buildings has not only changed the civil architecture, but also has created its own peculiar culture. In the current situation, ‘a place of one’s own’ has gradually lost its connotative meaning and as a result achieving a sense of ‘us’ seems to be a urgent and demanding task. That is, apartment dwelling culture has not yet been institutionalized and it is difficult to draw a distinct line between private, public and legal rights of citizens (2). On the other hand, residents of apartments have their own subcultures and are not in many cases familiar with the basics of apartment life. 

“Neighborhood problems” is a broad term that encompasses both physical and material features of the neighborhood as well as elements of social disorder (e.g. crime, loitering, street conflict or illegal drug use) (3). Researchers hypothesize that neighborhood problems may be a source of chronic stress that can contribute to poor mental health outcomes (4).

The concept of social solidarity (and the related concept of social cohesion, social capital) has gained very prominence in the social and public health literature in recent years although the opinion as far back as the 19th century (5-7). Social solidarity refers to the integration, and degree and type of integration, shown by a society or group with people and their neighbors. It refers to the ties in a society that bind people to one another (8).

Neighborhood social solidarity may influence psychosocial processes by providing individuals with a source of meaningful connection and mutual respect and increasing residents’ sense of purpose in life (9, 10).

All these make it imperative to know the culture and social values of apartment dwelling and its relations. The main objective of the present study was to investigate the relationship between social solidarity and mental health among apartment dwellers in Shahrekord, Iran.

## Materials and Methods

*Design and setting*

This cross sectional study was carried out in 2007 in Shahrekord, Iran. 

*Participants and sampling*

The participants were chosen from wives or husbands above the age of 18. Decision was also made to exclude residents with less than one year of stay in the neighborhood. The target population comprised of all apartment dwellers in Shahrekord among whom 200 were selected randomly for this study. For the determination of sample size, a 95% confidence interval and a 7% sample error were defined at first; the residential areas dense with apartment buildings were identified. Then, some blocks were randomly selected.

*Measures and Measurement*

The instruments used in this study were a checklist and two questionnaires which were supplemented with interviews. In checklist, demographic data including age, gender, education, occupation, and marital status, duration of residency in the neighborhood, total floor and apartment units were asked. Questionnaires included Fessler Social Solidarity Inventory (11) and General Health Questionnaire (GHQ) (12). 

Social solidarity questionnaire was translated to Persian and was modified according to the participants’ culture. In order to adapt the questionnaire to the realities of Iranian context, validity of the questionnaire as a data gathering instrument, was confirmed by selected experts in the fields of sociology, psychology and urban affairs and Cronbach's alpha coefficient (equal to 0.79) was calculated which confirmed its reliability. The final modified questionnaire had 35 items, with 5-scale Likert responses [from 5 (very good) to 1(very poor)]. Total score ranged between 35 and 175, in which lower the scores indicated lower solidarity state. Total scores were divided in 5 parts, 35- 63 as very poor, 64- 91 as poor, 92-119 as barely acceptable, 120-147 as good and 148-175 as very good. 

The GHQ is a measure of current mental health which was developed by Goldberg in 1970 (12). It has been extensively used in different settings and cultures. The questionnaire has also been standardized and contextualized according to the peculiarities of Iranian setting (13, 14). This instrument has been used in various studies so far and consists of 28 items. The answer to each item has four scales of 0: 'not at all', 1: 'same as usual', 2: 'more than usual' and 3: 'much more than usual'. The cut-off score was set at 23; all the lower scores were indicative of mental health and the higher scores indicated imbalance in the mental well-being.

*Codes of ethics*

Research Ethics Committee of Shahrekord University of Medical Sciences approved the study and all participants gave written informed consent. 

*Statistical analysis*

Analyses were performed using SPSS version 13. Data were described using mean and standard deviation (SD). For the analyses, statistical tests such as the Spearman correlation test and chi-squared and partial correlation test were used. P values less than 0.05 were considered significant.

## Results

Table 1 presents the demographic profile of 200 respondents. Mean (±SD) age of the participants was 30.9 (±8.8) years.

**Table 1:** Demographic profile of respondents (N=200)

	N	%
Female	100	50
Married	100	50
Education		
Less than high school	10	5
Some college + BA degree	140	70
Graduate training	50	25
No job or housewife	90	45
**Neighborhood characters**	Mean ± SD	
Number of years living in neighborhood	6.2 ± 6.4	
Number of units in apartment	7.3 ± 2.6	
number of total floor in apartment	4.7± 1.9	

*Social solidarity*

Social solidarity of the participants is presented in Table 2. We found significant relationship between social solidarity and duration of residency in neighborhood among the participants (p<0.05; r= 0.43). The relationship was also significant between age and social solidarity (p<0.05; r = 0.49). In addition, women showed a much better situation in terms of social solidarity compared to men when education and marital status were controlled (p<0.05). Besides, those with higher educational degrees enjoyed a higher level of social solidarity, yet this difference was not statistically significant (P>0.05).

**Table 2:** Social solidarity of the participants (N = 200)

	N	%
Very Good	40	20
Good	46	23
Barely Acceptable	36	18
Poor	40	20
Very Poor	38	19

*Mental Health*

Mean (±SD) mental health score of the sample was 17.1 (± 11.3). Overall, 31% of mental health scores found more than cut point. We could unravel significant relationship between gender and education with mental health. There was also a significant relationship between mental health scores of questioners and number of units in apartment (p<0.05; r = 0.51). Using partial correlation test and controlling the effect of age, sex and education, we found significant relationship between mental health and social solidarity (p<0.05; r = 0.47).

## Discussion

Our study showed that high social solidarity has positive relation with mental well-being of the participants. It demonstrated that buildings’ environment and neighborhood associated to mental health of the residents. Our study found duration of residency in the apartments was associated with improved social solidarity. Better social solidarity was associated with an increase in the age of the participants. We also found that poorer mental health of the participants was associated with an addition in the number of units in each apartment.

Our study supports previous finding suggesting an association between neighborhood social solidarity and mental health outcomes (4, 9, 10, 15). Matt et al. in a review also illustrated that poor psychosocial environments may be health damaging and contribute to health inequalities (16). In another study, Echeverria et al. found that individuals living in the least problematic neighborhoods were significantly less likely to be depressed, to smoke, or to drink. Less Socially cohesive neighborhoods were associated with increased depression, smoking, and not walking for exercise. Results persisted after adjusting for individual-level variables (17). 

The literature reports an association of advanced social solidarity with better mental health (18-21). Lee in a study on social solidarity showed the similar results that verifies the findings of our study (22). Another study also found that neighborhood social cohesion, measured by trust and reciprocity, is associated with higher self-rated health. However, social participation did not appear to be associated with better health in this predominantly low income neighborhood (23). The physical environment may have an indirect influence on mental health by changing psychosocial processes with known mental health consequences. Personal control, socially supportive relationships, and restoration from anxiety and exhaustion are all affected by features of the building environment. It is also likely that some individuals may be more mentally endangered by impacts of the building environment. 

Social isolation of mothers and limited playing opportunities for children are among some of the suspected reasons for the impact of high-rise apartments on psychological distress. In many high-rise apartments, particularly for low-income families, insufficient resources are assigned to spaces that are specified for the development and maintenance of social networks. 

Parents of young children in large multiple-unit apartments often deal with the lack of nearby playing spaces by keeping children inside their houses. Such restrictions increase intra familial conflicts, reduce play opportunities for children, and consequently make parents not so willing to get to know their neighbors. On the other hand, people feel better and have better mental health when they can control their environment (24).

There seems to be a direct relationship between social segregation factors or lack/low levels of social solidarity and the absence of the necessary skills for social interaction, lack of apartment management, the prevalence of diverse ethnic, linguistic, occupational and social subcultures, the paucity of daily interaction among dwellers due to their daily occupations, and the fact that many of the residents have rented their places and are therefore short-term residents, so as to make positive relationship between social solidarity and during of the residency in the apartments. Overall longer residency in the apartments causes the residents to get more acquainted with their neighbors and make them establish a consistent relationship with each other and adjust themselves to the current conditions of their apartments. 

It is unclear how representative our study is of the whole community; however, women had a better social solidarity status than men. Dunn et al. found gender differences in the relationship between housing, socioeconomic status, and self-reported health status (25).

Most probably, the higher social solidarity among women pertains to their higher levels of participation and therefore their increased interaction with other residents. Galab and Rao reported that the solidarity and unity among women members is one of the most important benefits as it gives them a forum to share their problems and seek help (26). In addition, higher education among residents, and as a result change in their perspective, seems to have positive impact on their social ties and their ability to adjust themselves to their context. This is achieved through their heavier supervision over their children conduct and their adaptation to the cultural specifics of living in apartment. 

Most of the participants in our study enjoyed an acceptable level of mental well-being. In Pollack et al. study in Germany about half of the participants had signs of mental problems. There is likely a relationship between mental well-being of the residents and the less number of the apartments and consequently fewer problems in their social environment (27).

Our study had some limitations. One limitation is that we did not measure sub scores of GHQ. An additional limitation was that our study design did not allow us to determine direction or the mechanism of the association between mental health and social solidarity.

## Conclusion

Based on obtained results, our hypothesis regarding association between social solidarity and mental health is confirmed. Longitudinal studies are needed to determine the effect of interventions targeting neighborhood and social relations on population's health.

## Authors’ Contributions

FG conceived and designed the evaluation and helped to draft the manuscript. AN and FS participated in designing the evaluation and performed the statistical analysis. NP revised the manuscript. ASh and HG participated in sampling of dissertation and collected the clinical data. All authors read and approved the final manuscript.

## Notes

**Declaretion of interest:** None.
